# Prediction of outcome in anal squamous cell carcinoma using radiomic feature analysis of pre-treatment FDG PET-CT

**DOI:** 10.1007/s00259-019-04495-1

**Published:** 2019-09-04

**Authors:** P. J. Brown, J. Zhong, R. Frood, S. Currie, A. Gilbert, A. L. Appelt, D. Sebag-Montefiore, A. Scarsbrook

**Affiliations:** 1 Department of Clinical Radiology, Lincoln Wing, Leeds Teaching Hospitals NHS Trust, St James’s University Hospital, Beckett Street, Leeds, LS9 7TF UK; 2 Radiotherapy Research Group, Leeds Institute of Medical Research at St James’s, Faculty of Medicine & Health, University of Leeds, Leeds, UK; 3 Department of Clinical Oncology, Bexley Wing, Leeds Teaching Hospitals NHS Trust, St James’s University Hospital, Beckett Street, Leeds, LS9 7TF UK

**Keywords:** Anal squamous cell carcinoma (ASCC), Radiomic feature analysis, FDG-PET/CT, Outcome prediction

## Abstract

**Purpose:**

Incidence of anal squamous cell carcinoma (ASCC) is increasing, with curative chemoradiotherapy (CRT) as the primary treatment of non-metastatic disease. A significant proportion of patients have locoregional treatment failure (LRF), but distant relapse is uncommon. Accurate prognostication of progression-free survival (PFS) would help personalisation of CRT regimens. The study aim was to evaluate novel imaging pre-treatment features, to prognosticate for PFS in ASCC.

**Methods:**

Consecutive patients with ASCC treated with curative intent at a large tertiary referral centre who underwent pre-treatment FDG-PET/CT were included. Radiomic feature extraction was performed using LIFEx software on baseline FDG-PET/CT. Outcome data (PFS) was collated from electronic patient records. Elastic net regularisation and feature selection were used for logistic regression model generation on a randomly selected training cohort and applied to a validation cohort using TRIPOD guidelines. ROC-AUC analysis was used to compare performance of a regression model encompassing standard clinical prognostic factors (age, sex, tumour and nodal stage—model A), a radiomic feature model (model B) and a combined radiomic/clinical model (model C).

**Results:**

A total of 189 patients were included in the study, with 145 in the training cohort and 44 in the validation cohort. Median follow-up was 35.1 and 37. 9 months, respectively for each cohort, with 70.3% and 68.2% reaching this time-point with PFS. GLCM entropy (a measure of randomness of distribution of co-occurring pixel grey-levels), NGLDM busyness (a measure of spatial frequency of changes in intensity between nearby voxels of different grey-level), minimum CT value (lowest HU within the lesion) and SMTV (a standardized version of MTV) were selected for inclusion in the prognostic model, alongside tumour and nodal stage. AUCs for performance of model A (clinical), B (radiomic) and C (radiomic/clinical) were 0.6355, 0.7403, 0.7412 in the training cohort and 0.6024, 0.6595, 0.7381 in the validation cohort.

**Conclusion:**

Radiomic features extracted from pre-treatment FDG-PET/CT in patients with ASCC may provide better PFS prognosis than conventional staging parameters. With external validation, this might be useful to help personalise CRT regimens in the future.

**Electronic supplementary material:**

The online version of this article (10.1007/s00259-019-04495-1) contains supplementary material, which is available to authorized users.

## Introduction

The incidence of anal cancer is rising in populations across the world [1–3]. This is mostly due to an increase in incidence of squamous cell carcinoma, the predominant histological type of anal cancer (ASCC). By comparison, adenocarcinoma, basaloid and cloacogenic histological types do not show the same increase in incidence [3]. Nonetheless, anal cancer remains a rare cancer with an incidence of 0.73 per 100,000 population [3]. The mainstay of treatment for non-metastatic ASCC is curative, non-surgical concurrent chemoradiotherapy (CRT), with only early anal margin tumours (stage T1 N0) suitable for local excision. CRT has been demonstrated to be the best curative treatment option for achieving local control, recurrence-free and/or progression-free survival (PFS) in ASCC compared with surgery or radiotherapy alone [4–7]. At present, this involves chemotherapy (mitomycin C and 5-fluorouracil) and concurrent radiotherapy, most commonly using 45–54 Gy in 1.8 Gy fractions depending on tumour stage [7].

The European Society for Medical Oncology (ESMO) Clinical Practice Guidelines recommend using multi-parametric magnetic resonance imaging (MRI) for accurate tumour staging and to inform radiotherapy treatment planning in ASCC [8, 9]. The guidelines also recommend use of baseline fluorine-18 fluorodeoxyglucose positron emission tomography/computed tomography (FDG-PET/CT) because of high sensitivity for identifying involved lymph nodes and distant metastases [9]. Systematic reviews report that FDG-PET/CT alters TNM stage in 41% of ASCC and influences a change in treatment plan in at least 28% of patients [10, 11]. Consequently, FDG-PET/CT is routinely performed as part of the initial imaging pathway at many institutions. Anal margin and anal canal tumour staging have been recently re-categorized (TNMv8), but, in both, local tumour (T) stage is predominantly determined by size [12]. Imaging features are combined with clinical assessment to provide a TNM stage and so risk categorise patients.

There is increasing interest in radiomics, which involves conversion of medical images into mineable high-dimensional quantitative data. The use of these data to predict treatment response and patient outcome has been reported across a range of primary tumours [13, 14]. There are very few studies evaluating radiomics in ASCC, but a recent study of 28 patients treated with curative-intent CRT reported that MRI texture analysis could predict tumour progression [15]. Other studies evaluating parameters derived from baseline FDG-PET/CT in ASCC patients have reported that metabolic tumour volume (MTV) [16, 17] and maximum standardized uptake value (SUV_max_) [18] predict local recurrence and overall survival. To the best of our knowledge, there are no studies evaluating FDG-PET/CT radiomics in ASCC or these measurements of tumour heterogeneity in combination with MTV, SUV_max_ and conventional prognostic factors (e.g. TNM stage) in a risk stratification prognostic model.

The aim of this study was to evaluate if radiomic features extracted from baseline FDG-PET/CT are predictive for PFS in patients with ASCC treated with curative-intent CRT compared with conventional staging. The secondary aim was to compare performance of a conventional prognostic feature model to a radiomic feature prognostic model and a combined model.

## Materials and methods

This study was designed as a transparent reporting of a multi-variable prediction model for Individual Prognosis or Diagnosis (TRIPOD) type 2 study designed to assess the potential benefit of FDG-PET/CT radiomics in patients with ASCC [19]. Adherence to this is detailed in Supplemental Table 1.

### Patient selection

Consecutive patients with histologically proven ASCC who underwent baseline FDG-PET/CT at a single large tertiary referral centre between June 2008 and 31st of December 2016 were identified retrospectively from a maintained database of FDG-PET/CT scans performed at our institution. Exclusion criteria included patients with small tumours when there had been pre-imaging excision of superficial lesions (total excision biopsies of tumours under 2 cm in size with a clear margin of at least 5 mm) or when lesions measured under 4 cm^3^. This is because there is a size threshold below which radiomic analysis may be unreliable and non-reproducible due to the delineation of the tumour [20, 21]. Furthermore, only patients treated with curative-intent CRT using standardised departmental protocols (concurrent radiotherapy, mean 49.6 Gy in 1.8-Gy fractions, with mitomycin C and 5-fluorouracil regimens) were included. Patients with advanced metastatic disease were therefore excluded as, in our institution, they received different treatment regimens.

Electronic clinical and radiological databases were used to obtain patient demographic details, clinical history, treatment data, clinical outcome and follow-up duration. The electronic records included the institutional radiology information system (Computerized Radiology Information System, (CRIS), Healthcare Software Systems, Mansfield, UK) and the oncology electronic patient record system (Patient Pathway Manager, PPM; EHR Development Team, Leeds Teaching Hospitals NHS Trust). The pertinent follow-up information included progression-free survival (PFS), comprising of locoregional failure (LRF), new distant metastatic disease and death (unless due to an alternative none ASCC cause of death, e.g. ruptured aneurysm), based on which occurred first with a median of 45-month clinical follow-up (interquartile range 28- to 61-month follow-up) [7, 22]. The LRF definition included all treatment failures or sites of disease recurrence occurring within the pelvis up to the level of the sacral promontory, either confirmed histologically by biopsy or where this was not possible by MDT consensus opinion [23].

Prospective consent was obtained from all patients at the time of imaging for use of their anonymised FDG-PET/CT imaging data in research and service development projects. All patients were prospectively entered into a departmental database used for retrospective identification and audit. Formal ethics committee approval was waived for this study which was considered by the institutional review board to represent evaluation of a routine clinical service.

### Radiomic feature analysis

Five steps were involved in ensuring objective radiomic feature analysis: image acquisition and reconstruction; image segmentation and rendering; feature extraction and quantification; databases and case sharing; ad hoc informatics analysis [24].

### Imaging acquisition and reconstruction

A standard protocol was used for FDG-PET/CT examinations with torso-imaging acquisition from the skull base to upper thighs. The CT component was acquired with the following settings: 140 kV; 80 mAs; tube rotation time 0.5 s per rotation; pitch 6; 3.75-mm section thickness. Patients were asked to maintain normal shallow respiration during the CT acquisition. No iodinated contrast material was administered. Serum blood glucose was routinely checked and if blood glucose was > 10 mmol/L, scanning was not performed. Patients fasted for 6 h prior to intravenous fluorine-18 FDG injection (dose varied according to patient body weight). Scans prior to June 2010 were performed on a 16-slice Discovery STE PET/CT scanner (GE Healthcare, Chicago, IL, USA) and from June 2010 to October 2015 on a 64-slice Philips Gemini TF64 scanner (Philips Healthcare, Best, Netherlands), After October 2015, all scans were performed on a 64-slice Discovery 710 scanner (GE Healthcare, Chicago, IL, USA). All scans used iterative reconstruction, CT for attenuation correction, applied scatter and randoms correction. Image reconstruction parameters for the different scanners are shown in Table 1. Each scanner used consistent reconstruction settings, matrix and voxel size.

**Table 1:** Reconstruction parameters for each scanner

Scanner	Reconstruction	Scatter correction	Randoms correction	Matrix	Voxel size (*x*, *y*, *z*)
GE Healthcare STE	OSEM	Convolution subtraction	Singles	128	4.6875 × 4.6875 × 3.27
Philips Gemini TF64	BLOB-OS-TF	SS-simul	DLYD	144 or 169	4 × 4 × 4
GE Healthcare Discovery 710	VPFX	Model based	Singles	192	3.65 × 3.65 × 3.27

*OSEM* ordered subsets expectation maximization, *BLOB-OS-TF* spherically symmetric basis function ordered subset algorithm, *VPFX* Vue Point FX (3D Time of Flight), *DLYD* delayed event subtraction

### Image segmentation and rendering

The entire segmentation and radiomic feature extraction process was performed using LIFEx software (v4.0, Local Image Feature Extraction, www.lifexsoft.org) [21].

The primary tumour and associated involved lymph nodes were delineated using a semi-automatic technique on PET/CT imaging by a single observer (clinical radiologist, 5-year experience) under supervision of an experienced dual-certified radiology and nuclear medicine physician (15-year experience of oncological PET/CT). A mean standardised uptake value was calculated in the right lobe of the liver (L-SUV_mean_) from a volume of interest (VOI) greater than 100 cm^3^ using a previously described method [25]. Using L-SUV_mean_ as a reference value, the primary tumour and associated involved lymph nodes were semi-automatically segmented to generate a tumour VOI (t-VOI) and separate lymph node VOIs (ln-VOIs). Voxels were included in the t-VOI or ln-VOI if they had an SUV greater than 1.5 times the L-SUV_mean_. This method generated more accurate VOIs than using a 40% SUV_max_ threshold that has been described elsewhere [26]. Lymph nodes were considered involved if they were enlarged (> 10 mm) and morphologically abnormal (rounded, soft tissue replacement of their fatty hilum and/or an irregular contour) in inguinal and/or iliac lymph node chains, and if they demonstrated SUV values greater than 1.5 times the L-SUV_mean_. Each t-VOI and LN-VOI was visually checked for accuracy and, where necessary, manually adjusted to exclude any non-tumour uptake. Special attention was paid to tumours located near the urinary bladder due to intense physiological urinary tracer activity and patients with background anal/GI tract FDG-activity. The same t-VOI and ln-VOIs were automatically segmented from the corresponding CT images.

Within each t-VOI, SUV and CT Hounsfield unit (HU) values were resampled into discrete bins using absolute resampling. This minimises the correlation between textural features and reduces the impact of noise and the size of matrices. Sixty-four bins were used for the PET component with the minimum and maximum bounds of the resampling interval set to 0 and 20 SUV; therefore, a bin size of 0.3 SUV was used for analysis of the PET component. Voxels with an SUV greater than 20 were grouped in the highest bin. For the CT component, voxels were resampled into 400 discrete bins across the range of − 1000 and 3000 HU; therefore, a bin size of 10 HU was used for the CT component analysis. Spatial resampling of the t-VOI and LN-VOI was performed using voxel dimensions of 4.0 × 4.0 × 4.0 mm for PET images and 2.5 × 1.2 × 1.2 mm (4.0 × 1.2 × 1.2 mm before June 2014) for CT images.

### Feature extraction

The feature extraction process used mathematical methods to evaluate the voxel intensity, their relative positions and the relationships between intensity and position to extract quantitative data from the t-VOI. The ln-VOI was only used to contribute to the total tumour volume and was not assessed by texture analysis. The radiomic texture analysis features are discussed in more detail elsewhere, vary in complexity based on the mathematical models they require, and all features extracted were based on standardised practices [21, 27]. In brief, first-order features extract information regarding either voxel intensity, with no spatial relationship information, or spatial information only with no intensity information included in their calculation. Second-order texture features compare relationships between adjacent voxels, whilst third-order texture features compare relationships between more than two voxels.

### Statistical analysis

All data was tabulated in Microsoft Excel (Office 365, 2017; Richmond, VA, USA) and statistical analysis was performed using SPSS (Version 16, 2016; IBM, Armonk, NY, USA), and RStudio (Version 1.1.134. RStudio: Integrated development environment for R. Boston, MA. http://www.rstudio.org/) using the glmnet package [28].

The study cohort was randomised on a ratio of 3:1 into ‘training’ and ‘validation’ cohorts using SAS (v9.4 SAS Institute Inc. Cary, NC, USA). Descriptive statistics (chi-squared and *t* test) were performed for the two cohorts and compared to ensure similarity between the groups. Elastic net regularisation was used for radiomic feature selection which automatically performs variable selection to shrink the model to reduce over fitting and co-variate correlation [29]. This technique has been shown to be superior to other methods of analysis when the set of features can be much larger than the number of cases [30]. To act as a comparator of current best practice, predicted outcomes were generated from the training cohort using a logistic-regression model (model A). This was based purely on standard clinical factors (patient age, sex, tumour and nodal stages), previously described in the literature as significantly related to oncological outcomes. Clinical factors (patient age, sex, tumour and nodal stages, planned radiotherapy dose and fractions) were included in the variable selection process alongside radiomic features. Two separate radiomic predictive models were created using radiomic features alone (model B) and combined with clinical features (model C).

The logistic-regression model based on elastic net feature selection and the model based on standard clinical features were separately developed on the training cohort and then tested on the validation cohort with predicted outcomes compared with PFS. Each set of predicted outcomes was compared with observed outcomes using receiver operating characteristic (ROC) analysis to assess each model’s ability to predict PFS.

## Results

Between 1st of June 2008 and 31st of December 2016, a total of 307 patients were identified for potential inclusion in the study. A total of 118 patients were excluded, reasons included; FDG-PET/CT imaging performed after excision of primary lesion—31 patients; FDG-PET/CT not performed—23 patients; non-ASCC histology—17 patients; treatment not administered with curative intent—13 patients; primary lesion too small for analysis (< 4 cm^3^, 64 voxels)—16 patients; primary tumour not visible on FDG-PET/CT—7 patients; incomplete imaging or clinical data—11 patients. After exclusions, there were 189 patients included in the study cohort.

The study cohort was randomised on a ratio of 3:1 into ‘training’ and ‘validation’ cohorts, containing 145 and 44 patients respectively. Detailed population descriptions are provided in Table 2. Within the total population, LN-VOI contributed only 0.35% to the median MTV volume (IQR 0.00–3.23%, median 0.07 cm^3^ [0.0–0.78 cm^3^]) with a median of one node per 3.3 patients (a maximum of 2 nodes were included per patient). A greater number of nodes were felt to be involved, and so staged as involved for clinically purposes but excluded from this analysis as they were too small or did not accumulate FDG.

**Table 2:** Population descriptions for the training, validation and combined total cohorts. Categorical data were compared between the cohorts using the chi-square test and continuous data was compared using the *t* test

Variable		Training cohort			Validation cohort			Total			Significance values
		*n* = 145			*n* = 44			*n* = 189			
		*n*	%	SD	*n*	%	SD	*n*	%	SD	
Sex	Male	46	31.7		16	36.4		62	32.8		0.566
	Female	99	68.3		28	63.6		127	67.2		
Mean age at diagnosis (years)		60.8		11.5	63.8		12.6	61.5		11.8	0.163
Tumour stage	T1	7	4.8		1	2.3		8	4.2		0.858
	T2	62	42.8		18	40.9		80	42.3		
	T3	43	29.7		15	34.1		58	30.7		
	T4	33	22.8		10	22.7		43	22.8		
Lymph node stage	N0	68	46.9		21	47.7		89	47.1		0.737
	N1	30	20.7		7	15.9		37	19.6		
	N2	30	20.7		12	27.3		42	22.2		
	N3	17	11.7		4	9.1		21	11.1		
Metastatic disease stage	M0	137	94.5		42	95.5		179	94.7		0.801
	M1	8	5.5		2	4.5		10	5.3		
Histology cell type	Basaloid squamous cell type	16	11.0		9	20.5		25	13.2		0.106
	Squamous cell carcinoma	129	89.0		35	79.5		164	86.8		
Histological grade	Low grade/well differentiated	18	12.4		4	9.1		22	11.6		0.922
	Moderate/medium differentiation	60	41.4		20	45.5		80	42.3		
	High grade/low differentiation	32	22.1		10	22.7		42	22.2		
	Not available	35	24.1		10	22.7		45	23.8		
Mean time interval from clinical diagnosis to FDG-PET/CT (days)		19.85		13.6	18.93		14.4	19.63		13.7	0.709
Mean injected FDG dose activity (MBq)		383.24		42.2	390.08		20.4	384.83		38.3	0.144
Mean fasting blood sugar at the time of FDG-PET/CT (mmol/L)		5.7		1.0	6.03		1.1	5.78		1.0	0.073
Mean SUV in the liver used as the reference for VOI determination		2.27		0.4	2.3		0.5	2.28		0.4	0.704
Mean time interval FDG-PET/CT to radiotherapy start date (days)		45.84		29.5	41.52		21.9	44.84		27.9	0.296
Mean time interval FDG-PET/CT to radiotherapy end date (days)		61.88		31.8	56.27		21.7	60.58		29.8	0.185
VMAT	No	114	78.6		35	79.5		149	78.8		0.895
	Yes	31	21.4		9	20.5		40	21.2		
Mean time from the start of radiotherapy until study censoring (months)		35.12		23.5	37.89		24.1	35.77		23.6	0.505
Locoregional failure (LRF)	No	108	74.5		32	72.7		140	74.1		0.816
	Yes	37	25.5		12	27.3		49	25.9		
Progression free survival	Yes (no LRF or metastatic disease)	102	70.3		30	68.2		132	69.8		0.784
	No	43	29.7		14	31.8		57	30.2		

*N* number of patients, *SD* standard deviation, *FDG-PET/CT* fluorine-18 fluorodeoxyglucose positron emission tomography/computed tomography, *SUV* standardized uptake value. *VOI* volume of interest, *VMAT* volumetric modulated arc therapy

**Fig. 1:**
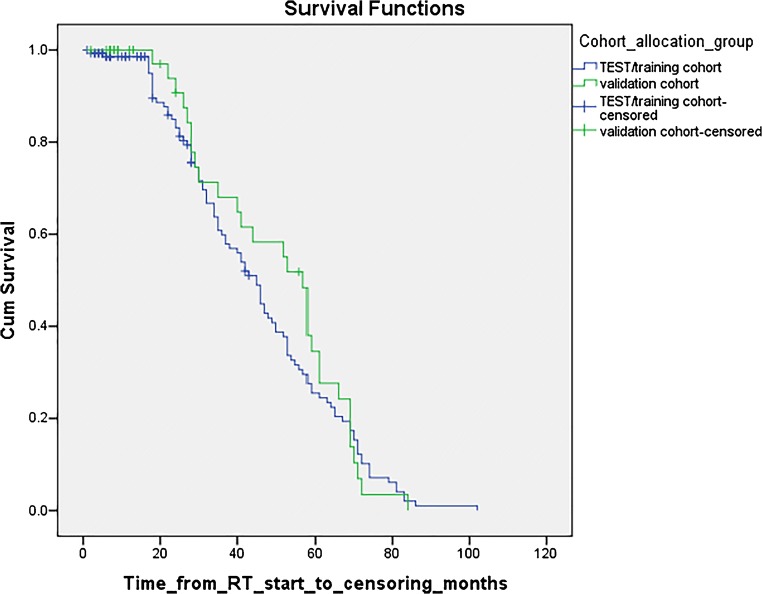
Kaplan-Meier survival curves comparing the progression-free survival between the training and validation cohorts. The log-rank between the two curves is 0.593, confirming no statistically significant differences between the cohorts

Likewise, each cohort had similar proportions of treatment failure and/or local disease recurrence; 37 patients (25.5%) in the training cohort and 12 patients (27.3%) in the validation cohort. The cohorts also had similar rates of non-local recurrence (distant sites of recurrence), 6 patients (4.1%) and 2 patients (4.5%) respectively. In the training cohort, 36 patients died whilst in the validation cohort 8 died; of these, at least 6 and 2 were due to non-ASCC causes (e.g. ruptured abdominal aortic aneurysm) and so included in the PFS group and censored at the time of their deaths. PFS was used as the outcome measure to incorporate absence of local and distant residual disease, delayed recurrence or new disease; again, similar PFS rates were demonstrated in each cohort (102 patients (70.3%) compared with 30 patients (68.2%) respectively), see Fig. 1. The log-rank between the two curves is 0.593, confirming no statistically significant difference between the cohorts. The median follow-up period for both groups was also similar at 35.12 months and 37.89 months from the start of radiotherapy to censoring, for the training and validation cohorts respectively.

The logistic regression model was calculated using the trial cohort and established variables reported to be of statistical significance in predicting outcome in ASCC including sex, age at diagnosis, tumour and lymph node stages. This model was similarly applied to the validation cohort. Elastic net regularisation obliviates the need for separate univariate analysis as the technique selects the variables for model inclusion as described in the “Materials and methods” section above. Using the training cohort, a mean cross-validated error value was plotted and a minimum value of 0.099 was calculated using elastic net regularisation for model B, and 0.190 for model C. Using this value as the minimum lambda value resulted in 10 variables being included in the prognostic model. These are documented in Table 3 and include conventional prognostic indicators (e.g. T and N stage), treatment details (radiotherapy dose and fractions) and radiomic features (e.g. grey-level co-occurrence matrix (GLCM) entropy and neighbourhood grey-level different matrix (NGLDM) busyness).

**Table 3:** Elastic net regularisation feature selection (model B)

Elastic net regularisation feature selection/model	Variable weighting
Tumour stage	− 0.011
Lymph node stage	− 0.019
Planned total radiotherapy dose (Gy)	0.007
Planned total radiotherapy fractions	0.012
Minimum CT value (HU)	0.000004
GLCM entropy log10- PET	− 0.002
GLCM entropy log2- PET	− 0.002
NGLDM busyness- PET	−0.023
Total SMTV (mL/Kg)	−0.037
Total TLG (SUV/mL)	−0.005
Constant	0.160

*CT* computed tomography, *PET* positron emission tomography, *HU* Hounsfield units, *GLCM* grey-level co-occurrence matrix, *NGLDM* neighbourhood grey-level different matrix, *SMTV* standardised metabolic tumour volume, *TLG* total lesion glycolysis

**Fig. 2:**
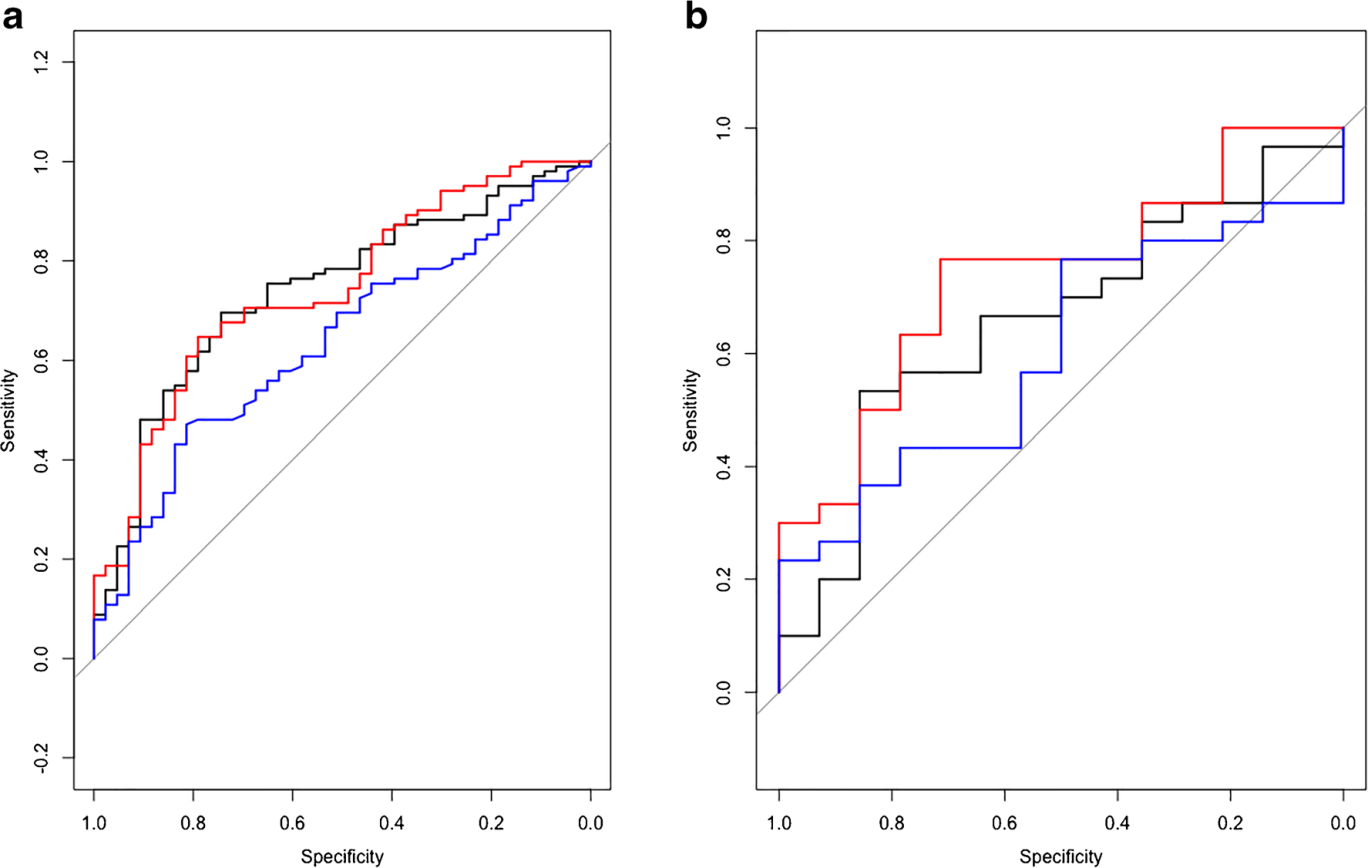
The blue line represents model A (clinical features), the black line represents model B (radiomic features) and red line represents model C (combined radiomic/clinical features), each generated on the training (**a**) and validation (**b**) cohorts. The AUCs for models A, B and C were 0.6355, 0.7403, 0.7412 for the training cohort and 0.6024, 0.6595, 0.7381 for the validation cohort

The prognostic elastic net regularisation model was applied to the validation cohort to generate predicted outcomes which were then compared with observed outcomes. Figure 2 demonstrates ROC curves generated for each model in the training (Fig. 2a) and validation (Fig. 2b) cohorts. The blue line represents model A, generated from clinical features only using a basic logistic regression technique. The black line represents model B generated from radiomic features alone using elastic net regression, and the red line represents model C generated from radiomic and clinical features using the same technique. The AUCs for models A, B and C were 0.6355, 0.7403, 0.7412 for the training cohort and 0.6024, 0.6595, 0.7381 for the validation cohort, respectively.

## Discussion

The results of this study indicate that radiomic features (objective measures of tumour heterogeneity) extracted from pre-treatment FDG-PET/CT may be useful to more reliably predict PFS in ASCC than standard features. In particular, the inclusion of GLCM entropy (a measure of the randomness of distribution of co-occurring pixel grey-levels), NGLDM busyness (a measure of the spatial frequency of changes in intensity between nearby voxels of different grey-level), the minimum CT value (lowest HU within the lesion) and a standardized version of MTV may provide superior, and more objective prediction of PFS than existing methods of prognostication. To our knowledge, this is the first study to report the potential of PET-derived radiomic feature analysis for outcome prediction in ASCC pre-treatment.

Pre-treatment risk modelling in ASCC is important; a current platform of three anal cancer trials (PLATO) is testing radiotherapy dose alteration in ASCC [23, 31]. In early-stage tumours, dose de-escalation is being evaluated (ACT4) and in locally advanced tumours does escalation (ACT5). The ability of the more reliable (and non-invasive) phenotype ASCC could be a valuable tool to further guide personalised treatment protocols for these tumours. Given the potentially serious patient morbidity associated with ASCC treatment, primarily radiation-related toxicity [32], accurate identification of patients with more aggressive tumour phenotype potentially warranting higher radiotherapy treatment doses is paramount. Improving imaging biomarkers is therefore important in ASCC to help offer more personalised radiation therapy [23]. The current study has shown that a model incorporating radiomic features extracted from FDG-PET/CT scans, acquired as part of routine clinical practice, can predict PFS with greater accuracy than existing methods. This compliments recent work by Hocquelet et al. in a small series of 28 patients reporting that MRI texture features were potential predictive biomarkers in ASCC [15].

MTV has previously been reported as a prognostic marker of overall survival in ASCC, with increasing tumour size or MTV correlating with poorer overall survival, either greater than 7 cm^3^ or greater than 26 cm^3^ [16, 33]. In the current study, the elastic net regularisation selected standardized MTV (SMTV—the MTV value relative to the patient’s body weight (cm^3^ kg^−1^) instead of MTV). Based on the principles of this modelling technique, the variables (MTV and SMTV) are likely to have been highly correlated and SMTV will have been selected because of its greater predictive power.

Similar to data reported by others, SUV_max_ was not a statistically significant predictor of progression-free survival [16]. However, total lesion glycolysis (TLG), a measure of SUV_mean_ relative to the size of a lesion (SUV_mean_/cm^3^), was of prognostic significance and selected for inclusion in the model. This was not included as a variable in the study by Gauthe et al., but was found to be a strong predictor of outcome in univariate analysis in the study by Bazan et al. [16, 33]. However, due to the correlation between TLG and MTV, the TLG was excluded from multivariate analysis [33]. Using SMTV, rather than MTV, will have decreased the correlation with TLG and so both variables were selected and included in the final model in our study.

Another, subtle difference of note is the definition of MTV. Here, MTV (and therefore SMTV and by extension TLG) incorporated the sum of t-VOI and LN-VOI, as did Bazan et al. [33]. This was considered more representative of the entire tumour volume than t-VOI alone, as used by Gauthe et al. [16]. However, on review, the LN-VOI contributed only 0.35% to the median MTV volume (IQR 0.00–3.23%, median 0.07 cm^3^ [0.0–0.78 cm^3^]); therefore, this distinction is most likely arbitrary unless the burden of lymph node disease is significantly greater than the primary lesion itself.

The variables selected by the elastic net regularisation model are all features providing a measure of tumour heterogeneity. This included the minimum CT value (HU) which it is postulated maybe because tumours with a worse prognosis are more likely to have increased intra-lesion degeneration and necrosis resulting in intra-lesion gas locules [34].

The retrospective nature of this study is a limitation, but the low incidence of ASCC and high PFS rates, relative to other cancers, make it more challenging to acquire large prospective data. Furthermore, the exclusion of very small and advanced metastatic tumours further limited the inclusion criteria to only those tumours suitable for CRT administered with curative intent. Nonetheless, we have analysed a relatively large patient cohort treated with standardised departmental protocols. Additionally, a standardised imaging protocol was used throughout the study period, despite three different PET/CT scanners being used, and the random cohort allocation prior to analysis has ensured as robust a methodology as possible to overcome this issue. Furthermore, spatial resampling and intensity binning performed on all data increases adherence to key methodological principles of radiomics and the repeatability of this study [35–39].

The spatial resolution of pelvic MRI is superior to that of CT and/or PET imaging. However, MRI scans were not analysed in this study because of a lack of consistency in the imaging protocols and scan acquisition parameters in clinical use. As a result, image signal intensity values can show significant variability across different patients, scanners and protocols, inherently restricting the usefulness of radiomic feature analysis. By comparison, the intensities of voxels in PET and CT images have been studied to a greater degree and are more reliable, assuming the use of a robust intensity binning method [40–42]. Whilst an additional harmonisation step to further improve the reliability of PET/CT derived radiomic features has recently been reported, following data collection and analysis had been completed for this study, and no similar harmonisation method is established for MRI [43, 44]. In the absence of widely accepted MRI harmonisation process, the use of a single MRI scanner/protocol for all patients may have minimised the impact of some inconsistencies in MRI signal, but it would also significantly limit the clinical impact of any findings. This warrants further study.

Another potential limitation is only one observer performing the tumour segmentation. However, as the segmentation was performed semi-autonomously, the observer input had already been minimised which will have helped to mitigate potential intra- and inter-observer differences. External validation of the findings in this study is required in the first instance to ensure the results are reproducible and/or require refinement. Following this, incorporation of the methodology into a future well-designed multi-centre prospective trial would be required in order to confirm benefit in routine patient management.

## Conclusions

Radiomic features extracted from pre-treatment FDG-PET/CT in patients with ASCC may provide greater accuracy in predicting PFS than conventional staging parameters. This could have a potential powerful clinical impact by helping risk stratify and personalise treatment in ASCC patients. External validation of the results in this initial study, and prospective evaluation in a multi-centre cohort, is required before a clear impact on clinical decisions can be confirmed.

## Electronic supplementary material

Supplemental Table 1 (DOCX 103 kb)
