# Supplementary material for: Prediction of outcome in anal squamous cell carcinoma using radiomic feature analysis of pre-treatment FDG PET-CT
Source: Eur J Nucl Med Mol Imaging. 2019 Sep 4;46(13):2790–9. doi: 10.1007/s00259-019-04495-1 (PMC6879433; doi:10.1007/s00259-019-04495-1)
Supplement: Supplementary file 1 — (DOCX 103 kb) [file 259_2019_4495_MOESM1_ESM.docx]

| **Section/Topic** | **Item** | **Checklist Item** | **Page** |
| --- | --- | --- | --- |
| **Title and abstract** | | | |
| Title | 1 | Identify the study as developing and/or validating a multivariable prediction model, the target population, and the outcome to be predicted. | 1 |
| Abstract | 2 | Provide a summary of objectives, study design, setting, participants, sample size, predictors, outcome, statistical analysis, results, and conclusions. | 2 |
| **Introduction** | | | |
| Background and objectives | 3a | Explain the medical context (including whether diagnostic or prognostic) and rationale for developing or validating the multivariable prediction model, including references to existing models. | 3 |
|  | 3b | Specify the objectives, including whether the study describes the development or validation of the model or both. | 3 |
| **Methods** | | | |
| Source of data | 4a | Describe the study design or source of data (e.g., randomized trial, cohort, or registry data), separately for the development and validation data sets, if applicable. | 4, 6 |
|  | 4b | Specify the key study dates, including start of accrual; end of accrual; and, if applicable, end of follow-up. | 4 |
| Participants | 5a | Specify key elements of the study setting (e.g., primary care, secondary care, general population) including number and location of centres. | 4 |
|  | 5b | Describe eligibility criteria for participants. | 4 |
|  | 5c | Give details of treatments received, if relevant. | 4 |
| Outcome | 6a | Clearly define the outcome that is predicted by the prediction model, including how and when assessed. | 4 |
|  | 6b | Report any actions to blind assessment of the outcome to be predicted. | * |
| Predictors | 7a | Clearly define all predictors used in developing or validating the multivariable prediction model, including how and when they were measured. | 4, 5, 6 |
|  | 7b | Report any actions to blind assessment of predictors for the outcome and other predictors. | * |
| Sample size | 8 | Explain how the study size was arrived at. | * |
| Missing data | 9 | Describe how missing data were handled (e.g., complete-case analysis, single imputation, multiple imputation) with details of any imputation method. | 7 |
| Statistical analysis methods | 10a | Describe how predictors were handled in the analyses. | 6, 7 |
|  | 10b | Specify type of model, all model-building procedures (including any predictor selection), and method for internal validation. | 6, 7 |
|  | 10d | Specify all measures used to assess model performance and, if relevant, to compare multiple models. | 7 |
| Risk groups | 11 | Provide details on how risk groups were created, if done. | N/A |
| **Results** | | | |
| Participants | 13a | Describe the flow of participants through the study, including the number of participants with and without the outcome and, if applicable, a summary of the follow-up time. A diagram may be helpful. | 7 |
|  | 13b | Describe the characteristics of the participants (basic demographics, clinical features, available predictors), including the number of participants with missing data for predictors and outcome. | Table 2 |
| Model development | 14a | Specify the number of participants and outcome events in each analysis. | Table 2 |
|  | 14b | If done, report the unadjusted association between each candidate predictor and outcome. | N/A |
| Model specification | 15a | Present the full prediction model to allow predictions for individuals (i.e., all regression coefficients, and model intercept or baseline survival at a given time point). | Table 3 |
|  | 15b | Explain how to use the prediction model. | Table 3 |
| Model performance | 16 | Report performance measures (with CIs) for the prediction model. | Figure 2 |
| **Discussion** | | | |
| Limitations | 18 | Discuss any limitations of the study (such as nonrepresentative sample, few events per predictor, missing data). | 13, 14 |
| Interpretation | 19b | Give an overall interpretation of the results, considering objectives, limitations, and results from similar studies, and other relevant evidence. | 13, 14 |
| Implications | 20 | Discuss the potential clinical use of the model and implications for future research. | 13, 14 |
| **Other information** | | | |
| Supplementary information | 21 | Provide information about the availability of supplementary resources, such as study protocol, Web calculator, and data sets. | 5, 6 |
| Funding | 22 | Give the source of funding and the role of the funders for the present study. | N/A |

*See comments

We recommend using the TRIPOD Checklist in conjunction with the TRIPOD Explanation and Elaboration document.

1. This study looks at a prediction model using radiomic features of PET/CT in anal squamous cell carcinoma. (**Title**)
2. The abstract covers a summary of all the requested information. (**Abstract**)
3. a) The introduction presents the background of ASCC and sets out the aim of creating a predictive model using radiomic and conventional features. As far as the authors are aware no predictive models using these features exist currently and therefore none are discussed. (**Introduction**)

b) The aim of study is to develop a prognostic model using radiomic and conventional features. (**Introduction**)

1. a) This is a retrospective cohort study with the methodology described in paragraph 1 of the patient selection section. The study cohort was randomised on a ratio of 3:1 into ‘training’ and ‘validation’ cohorts using SAS to create an internal validation. (**Patient selection**)

b) Consecutive patients with histologically proven ASCC who underwent baseline FDG-PET/CT at a single large tertiary referral centre between June 2008 and 31st December 2016 were included. The pertinent follow-up information included in the study is also set out in this section. (**Patient selection**)

1. a) As stated this is a single tertiary centre study. (**Patient selection**)

b) Only patients treated with curative-intent CRT using standardised departmental protocols. Patients with lesions which had been excised pre-imaging and patients with lesions under 4cm^3^ were excluded. (**Patient selection**)

c) The treatment regimen as set out by the standard departmental protocol is documented. (**Patient selection**)

1. a) The outcomes are defined. (**Patient selection**)

b) As this was a retrospective study the primary outcomes were defined from clinical records. The investigator reviewing the records was blinded to the imaging parameters.

1. a) A detailed description of the radiomic data extraction is outlined in the methods section as well as reference to the radiomic parameters which were extracted. The features selected for the model via the elastic net regulation method are documented in table 3. The clinical parameters are also documented within the method section. (**Materials and methods**)

b) The images were contoured and analysed without reference to the outcome data.

1. Due to the nature of ASCC, a limited number of patients were available. It was therefore not feasible to have a sample size large enough to create a model to achieve the accepted 10 events per parameter required for a standard logistic regression model. Based on the 53 suggested parameters and the reported recurrence rate of 26%, the expected sample size required would be >2000. Therefore, elastic net regularisation was incorporated into the study. This has been demonstrated to be an effective method for parameter selection in studies when parameters greatly outnumber sample size e.g. genetic assay studies.
2. Only complete data sets were used in the analysis. 11 patients were excluded due to incomplete imaging or clinical data. (**Results**)
3. a) Clinical factors (patient age, sex, tumour and nodal stages, planned radiotherapy dose and fractions) were included in the variable selection process alongside radiomic features. To compare with current practice, a logistic-regression model based purely on standard clinical factors (patient age, sex, tumour and nodal stages) was also performed. (**Statistical analysis**)

b) Elastic net regularisation was used for radiomic feature selection which automatically performs variable selection to shrink the model to reduce over fitting and co-variate correlation. (**Statistical analysis**)

d) Receiver operating characteristic (ROC) analysis was performed to assess each model’s ability to predict PFS. (**Statistical analysis**)

1. Risk groups were not created within the model.
2. a) Between 1^st^ June 2008 and 31^st^ December 2016, a total of 307 patients were identified for potential inclusion in the study. After exclusions there were 189 patients included in the study cohort. (**Results**)

b) The characteristics of the participants are presented in **Table 2**.

1. a) The number of events per model are presented in **Table 2**.

b) This has not been performed. Univariate analysis was performed to demonstrate no difference between parameters in the training cohort and internal validation cohort.

1. a/b) The variable weightings and constant used in the model are demonstrated in **Table 3.**
2. ROC curves with AUCs are presented, demonstrating the performance of the models. (**Figure 2**)
3. The limitations of the study are presented. These include the retrospective nature of the study, the low incidence of ASCC, small lesions being excluded and a single observer performing tumour segmentation. (**Discussion**)
4. b)/20. The discussion section gives an overall interpretation of the results and highlights the potential use of a pre-treatment model to aid in personalised treatment for patients. (**Discussion**)
5. Links to the radiomics software used ([www.lifesoft.org](http://www.lifesoft.org)) and elastic net regression software (<http://www.rstudio.org/>) are included. (**Materials and methods**)
6. The study was not externally funded. There are no conflicts of interest to declare as set out in the journal declaration. (**Declaration**)
